# Classifying Hyponatremias According to Tonicity Disorder: Hypotonic Hyponatremia, Hypertonic Hyponatremia, and Pseudohyponatremia as Distinct Entities

**DOI:** 10.7759/cureus.95696

**Published:** 2025-10-29

**Authors:** Todd S Ing, Raymond E Garrett, Ramin Sam, Christos Argyropoulos, Susie Q Lew, Maria-Eleni Roumelioti, Mark Unruh, Darlene Vigil, Mark Rohrscheib, Brent Wagner, Antonios H Tzamaloukas

**Affiliations:** 1 Medicine, Stritch School of Medicine, Loyola University Chicago, Maywood, USA; 2 Trauma Research, Swedish Medical Center, Englewood, USA; 3 Nephrology, University of California San Francisco, San Francisco, USA; 4 Nephrology, University of New Mexico School of Medicine, Albuquerque, USA; 5 Medicine, George Washington University, Washington, D.C., USA; 6 Nephrology, Raymond G. Murphy Department of Veterans Affairs Medical Center, Albuquerque, USA; 7 Research, New Mexico Veterans Affairs Health Care System, Albuquerque, USA

**Keywords:** hypertonic hyponatremia, hypotonic hyponatremia, osmol gap, pseudohyponatremia, serum osmolality, serum osmolarity, serum tonicity

## Abstract

Tonicity of a solution refers to its ability to cause cells in it to either swell or shrink through osmotic fluid flux across the cell membranes due to a difference in the total concentrations of osmoles in the solution and in the intracellular space. For serum, the sodium concentration ([Na]_S_) constitutes the major determinant of tonicity. Three categories of tonicity can be found in cases of hyponatremia: (a) hypotonic hyponatremia created by a relative excess of body water over the sum of exchangeable body sodium plus exchangeable body potassium and causing osmotic fluid entry into cells; (b) hypertonic hyponatremia created by gain of an extracellular solute other than a sodium salt causing osmotic fluid exit from cells; and (c) pseudohyponatremia (isotonic hyponatremia), which is the result of measurement of [Na]_S_ by a method requiring pre-measurement dilution of the serum sample in patients with a high serum solid content. In this latter case, the low value of the measured [Na]_S_ falsely suggests hypotonic hyponatremia. The principles of treatment vary among these three categories of hyponatremia. Identifying the pathophysiological category of a case of hyponatremia constitutes the first step in its management. This review presents important clinical characteristics of the three pathophysiological categories of hyponatremia and a method for identifying the category of any hyponatremia.

## Introduction and background

A cell's volume is very important for its functions and survival. Key mechanisms for keeping this volume constant consist of extrusion of solutes by active processes into the extracellular compartment and high permeability of the cell membranes to water [1]. In the steady state, the osmolalities of the intracellular and extracellular fluids of cell membranes with high water permeability are equal. The cell membranes of the vast majority of the body cells contain aquaporins and have a high permeability to water. The intracellular fluids contain substantially higher concentrations than the extracellular fluids of polyanions, e.g., proteins, that do not cross the cell membranes. The intracellular polyanions electrostatically attract electrolyte cations and drive electrolyte anions through the cell membranes. The Gibbs-Donnan equilibrium expresses the relationship between the electrolyte anions and cations in the fluids across a membrane that contains polyanions in one fluid. The mathematical expression of this equilibrium dictates that the sum of electrolyte anions and cations is higher in the fluid containing the polyanions [1]. The high permeability of cell membranes to water would cause water entry into the cells because of the Gibbs-Donnan equilibrium. This is prevented by the continuous extrusion of electrolyte cations from the cells. Sodium extrusion from the cells in excess of potassium entry into the cells by the energy-consuming action of the sodium-potassium ATPase of cell membranes is a key mechanism protecting the volume of intracellular fluid [1].

The major clinical conditions causing changes in the cell volume are disturbances of the tonicity (effective osmolarity) of the extracellular fluid [1]. The tonicity of a solution is its property to reduce, not change, or increase the volume of cells suspended in it through osmotic water transfer across cell membranes. The clinical application of the serum sodium concentration ([Na]_S_) is its use as an indicator of the tonicity of the extracellular fluid [2,3]. Every case of hyponatremia, defined as [Na]_S_ < 135 mmol/L, should be classified and approached clinically according to the change in serum tonicity that it indicates.

There are three distinct pathophysiological categories of hyponatremia in terms of the changes in the intracellular volume of cells caused by the tonicity of the serum, or more precisely, of the interstitial fluid connected to this serum: hypotonic hyponatremia, hypertonic hyponatremia, and pseudohyponatremia [4,5]. It is important to identify the category of tonicity indicated by a low [Na]_S_ because both the investigation for the pathogenesis and the management vary between the three categories of hyponatremia. Deciding whether a correction of the intracellular volume is required constitutes the first step of the clinical management of any case of hyponatremia. If such a correction is required, the next step is to define whether the cell volume is increased or reduced.

The main aim of this report is to present a method for identifying the tonicity category of any case of hyponatremia. Synoptic descriptions of each category of hyponatremia are also presented. Details of the pathogenesis and treatment methods of these categories can be found in the references cited. These references were obtained from PubMed. References presenting the pathophysiological concepts of cell volume regulation and abnormalities, measurement issues relevant to the three categories of hyponatremia, and pitfalls in the diagnosis of hyponatremia were selected. The PubMed search terms included hyponatremia, hypotonic hyponatremia, true hyponatremia, hyponatremia guidelines, hypertonic hyponatremia, hyperosmolar hyponatremia, pseudohyponatremia, isotonic hyponatremia, and specific treatments, e.g., vaptans in hyponatremia.

## Review

Hypotonic hyponatremia

Definition and Pathophysiology

Classification of a case of hyponatremia to the hypotonic category indicates that the patient's cells are swollen through osmotic transfer of water. Sodium concentration in serum water and serum osmolality are two key tonicity indices that should be evaluated in all cases of dysnatremia [2]. The Edelman study established a strong statistical association between these two indices [6]. According to this study, hypotonic hyponatremia results from decreases in the fraction {exchangeable total body sodium (ETBNa) + exchangeable total body potassium (ETBK)}/{total body water (TBW)} due to single or combined losses or gains of body sodium, potassium, and water [6].

Hypotonic hyponatremia is the most common electrolyte abnormality [7] and is associated with increased morbidity and mortality [8]. The general mechanisms of hypotonic hyponatremia include increased water intake and decreased urinary excretion of water, which can result from inability to dilute the urine, low glomerular filtration rate, or low urine solute load [5]. The presence of one mechanism or combinations of mechanisms should be investigated by the history and physical examination of the patient and by measurements of urine volume, osmolality, sodium, and potassium concentrations, and in some cases by specific laboratory tests in the serum [5]. The key clinical manifestations of hypotonic hyponatremia are consequences of osmotic brain cell swelling [5,7].

Management Principles

Several guidelines and reports have addressed the correction of hypotonic hyponatremia [9-15]. The steps of the management of this condition consist of identifying the mechanism that led to an abnormally low fraction $(ETBNa + ETBK)/TBW$, determining whether body water and extracellular volume are low, normal, or above normal and whether the hyponatremia is acute or chronic, applying the proper methods for its correction, and carefully monitoring the clinical features and the relevant laboratory values of the patient during and after the treatment period [3-5,9-15].

The duration of hyponatremia affects both the severity of its clinical manifestations and its management. Hypotonic hyponatremia should be categorized into one of two stages, acute or chronic, depending on the changes in brain organic osmolytes at presentation [16]. Whether a case of hyponatremia is acute or chronic should be considered in determining its correction rate [16]. When sequential measurements of the [Na]_S_ are not available, the assignment of a case of hyponatremia to the acute or chronic category is based on the best clinical information accessible [11].

The methods for treating hypotonic hyponatremia consist of measures for increasing [Na]_S_ and correcting the conditions resulting in it. The fluid and monovalent cation management aims to return the fraction $(ETBNa + ETBK)/TBW$ within the normal range. Infusion of hypertonic saline is necessary in cases of hyponatremia with severe clinical manifestations [11,17]. Several formulas have been reported for the calculation of the volume of hypertonic saline required to produce a prescribed rise in [Na]_S_ [18]. The reported formulas have potential deficits [19]. In several studies, the average rises in [Na]_S_ in studies using these formulas agreed in general with the prediction of the formulas, but the ranges of these rises were wide [18]. The general treatment measures address restriction of fluid intake and increase of free water clearance by loop diuretics and/or administration of solutes excreted in the urine, including salt tablets and urea [5].

Examples of measures to treat specific conditions leading to hypotonic hyponatremia include the following: (1) Administration of vasopressin receptor inhibitors [20], used primarily for the management of the syndrome of inappropriate antidiuretic hormone secretion (SIADH) or hypervolemic hyponatremia, e.g., in congestive heart failure [21]. In these conditions, water retention secondary to high plasma levels of antidiuretic hormone (ADH) is the cause of hyponatremia. (2) Desmopressin infusion, which should be used to prevent a rapid increase of the [Na]_S_ during the treatment of hyponatremias associated with low serum ADH levels and dilute urine [15,20,22,23]. A systematic review [24] and two subsequent retrospective studies [25,26] concluded that prospective studies are needed to establish the benefits and risks of this use of vasopressin. (3) Hormone replacement therapy in hyponatremias associated with specific hormonal deficiencies, e.g., hypothyroidism or adrenal insufficiency [5]. (4) Diagnosis and treatment of the condition causing SIADH [27].

Hypertonic hyponatremia

Definition and Pathophysiology

Hypertonic hyponatremia is the consequence of osmotic transfer of water out of cells. The pathophysiological principle governing the development and the correction of hypertonic hyponatremia states that the amounts of solute in the intracellular and extracellular compartments determine the distribution of body water between these two compartments [28]. This principle is expressed as (intracellular water)/(extracellular water) = (intracellular solute)/(extracellular solute) [29]. Hypertonic hyponatremia, also known as translocational or hyperosmolar hyponatremia, develops after a gain of any solute with extracellular distribution other than a sodium salt. An isolated gain in extracellular solute decreases the fraction (intracellular solute)/(extracellular solute), causing osmotic fluid transfer from the intracellular into the extracellular compartment. The sodium in this latter compartment is diluted when the solute gained is other than a sodium salt. Extracellular solutes causing hypertonic hyponatremia are either exogenous or endogenous.

Exogenous Solutes

Examples of exogenous solutes causing hypertonic hyponatremia include mannitol, maltose, sucrose, glycine, sorbitol, and icodextrin. Hypertonic mannitol infusions are used to correct brain cell edema in traumatic brain injury [30,31]. Hypertonic hyponatremia after infusion of preparations of immunoglobulins containing maltose [32] or sucrose [33] is combined with pseudohyponatremia caused by the high serum protein levels (see *Pseudohyponatremia* below). Absorption into the circulation of hypotonic or isotonic glycine or sorbitol solutions used to irrigate the operative field of transurethral prostatectomy or uterine surgery results in a combination of hypertonic and hypotonic hyponatremia [34]. A clue to this combination of hyponatremias is provided by the serum osmolality, which is normal or slightly low, while the [Na]_S_ is substantially low [35]. Hypertonic hyponatremia results from the absorption from the peritoneal cavity into the blood of icodextrin, an osmotic agent used in peritoneal dialysis. Icodextrin and its metabolic products, including maltose and other oligosaccharides, accumulate in the extracellular fluid [36].

Endogenous Solute

The hyperglycemic syndromes are, by far, the main causes of hypertonic hyponatremia [29]. The pathophysiology and treatment of disturbances in both body fluids and [Na]_S_ differ between hyperglycemias occurring in patients with preserved renal function and those with oligo-anuria. Patients in this latter category exemplify the tonicity changes produced exclusively by the gain of extracellular solute in hyperglycemia. These patients present routinely with hypertonicity and hyponatremia and can be treated solely with insulin infusion. No other measures are usually needed, as there are no losses or gains of fluid or electrolytes. In most patients in this category, [Na]_S_ returns within its usual normal range after correction of the hyperglycemia [29,37].

Severe hyperglycemic syndromes in patients with preserved renal function routinely produce profound deficits of fluid and electrolytes through osmotic diuresis, causing combined states of extracellular volume depletion and dysnatremias [29,38,39]. The deficits of water and extracellular volume may vary greatly between hyperglycemic patients with the same values of serum glucose ([Glu]_S_) and [Na]_S_ [40]. The value of [Na]_S_ that would be obtained by the correction of [Glu]_S_ to 5.6 mmol/L (100 mg/dL) without changes in body water or solutes other than glucose ([Na]_Cor_) is the best index of the level of dehydration in these patients [41], who with hyperglycemia may have [Na]_S_ values ranging from hyponatremia to hypernatremia [40]. [Na]_Cor_ should be computed by the formula $[Na]Cor = [Na]S + (1.6 x ({[Glu]S - 5.6}/5.6)$ [41]. The degree of hypovolemia should be calculated using [Na]_Cor_ and estimates of the volume of body fluids lost and of the concentrations of sodium and potassium in the lost fluids [42].

Pseudohyponatremia

Definition and Pathophysiology

Pseudohyponatremia (also known as false hyponatremia) is a false categorization of sodium concentration in serum water ([Na]_SW_) as low by [Na]_S_. In the pure cases of pseudohyponatremia, [Na]_SW_ and cell volume are normal. [Na]_S_, [Na]_SW_, and sodium concentration in interstitial fluid water ([Na]_IFW_) vary. [Na]_IFW_, which is not measured in clinical practice, would be the most appropriate index of tonicity because the interstitial fluids are in direct contact with the cells. The relationship between [Na]_IFW_ and [Na]_SW _is determined by the Gibbs-Donnan equilibrium, which dictates that the concentrations of cations crossing a semipermeable membrane are higher in the compartment that contains higher concentrations of non-permeable anions [43]. The plasma proteins with pK values lower than the normal pH of the blood, mainly albumin, which has a pK of 4.8, are dissociated into polyanions and protons, while the interstitial fluids on the other side of the membranes separating them from the plasma contain very low concentrations of polyanions. Due to the Gibbs-Donnan equilibrium, [Na]_SW_ has a higher value than [Na]_IFW_. Still, in most instances, the difference between the two sodium concentrations is relatively small, making [Na]_SW_ a reliable index of extracellular fluid tonicity. One notable exception is the presence of cationic paraproteins in the sera of hematologic malignancies. Low or negative values of the serum anion gap are a consequence of serum cationic proteins [44,45]. According to the Gibbs-Donnan equilibrium, the [Na]_IFW_ is higher than the [Na]_SW_ when the serum anion gap is negative. This may have an adverse effect on the accuracy of [Na]_SW_ as an index of tonicity.

The clinical laboratories report [Na]_S_. The relation between [Na]_S_ and [Na]_SW_ is the source of pseudohyponatremia. The serum has a water fraction and a solid fraction. Sodium and other dissolved crystalloids are contained in the water fraction of the serum. The solid fraction, consisting of proteins and lipids, contains no sodium. [Na]_SW_ is always higher than [Na]_S_. However, the difference between [Na]_SW_ and [Na]_S_ varies depending on the relation between serum water content (SWC) and serum solid content (SSC) [46-48]. SWC and SSC are expressed as parts of one: the normal value of SWC is 0.93, and the normal value of SSC is 0.07 [48]. At a [Na]_SW_ of 154 mmol/L, [Na]_S_ in mmol/L is 143.2 (154x0.93) at an SWC of 0.93, 135.5 (154x0.88) at an SWC of 0.88, and 150.2 (154x0.98) at an SWC of 0.98.

[Na]_S_ is measured by methods either requiring pre-measurement dilution of the serum sample (flame photometry or indirect ion-specific potentiometry) or not requiring pre-measurement dilution (direct potentiometry). Most main hospital laboratories measure [Na]_S_ by indirect potentiometry [48]. The autoanalyzers using potentiometry methods measure the electrical potential, which directly reflects the [Na]_SW_ of the diluted or non-diluted serum sample. The algorithms of the autoanalyzers convert to [Na]_S_ the measured electrical potential, assuming an SWC of 0.93 [48]. When SWC is lower than 0.93, indirect potentiometry reports a [Na]_S_ value close to the actual value but provides false information about [Na]_SW_ [48]. This condition has been called pseudohyponatremia, spurious hyponatremia, or isotonic hyponatremia because the [Na]_SW_ is within the normal range in its pure forms.

The intracellular volume of the cells suspended in serum with low SWC and [Na]_S_, but with normal [Na]_SW_, remains normal. In this case, measurement of [Na]_S_ by direct potentiometry provides a normal value [48]. Although this normal value of [Na]_S_ is false because the concentration of sodium in serum is low, it provides indirectly correct information about [Na]_SW_ [48]. [Na]_S_ measurements by indirect potentiometry provide falsely high estimates of [Na]_SW_, i.e., pseudonormonatremia or pseudohypernatremia, at SWC higher than 0.93, which are encountered in cases of hypoproteinemia [49]. Note that true hyponatremia should be defined as a low [Na]_SW_ value. By this definition, both hypertonic hyponatremia [50] and hypotonic hyponatremia are true hyponatremias, while pseudohyponatremia is not.

Conditions Associated With Pseudohyponatremia 

Pseudohyponatremia has been reported in patients with low SWC secondary to hyperproteinemia, hypertriglyceridemia, and hypercholesterolemia [48]. Clinicians should be aware of the method used to measure [Na]_S_. Pseudohyponatremia should be considered in cases of hyponatremia measured by indirect potentiometry. If hyperproteinemia or hyperlipidemia is suspected or documented, repeated measurement of [Na]_S_ by direct potentiometry should be performed.

Pseudohyponatremia Management and Future Developments

Arterial blood gas apparatuses can measure [Na]_S_ by direct potentiometry [48]. Measuring [Na]_S_ by direct potentiometry or by one of the newer methods [48] will eliminate almost all cases of pseudohyponatremia. The exceptions consist of cases not associated with low SWC, e.g., pseudohyponatremia observed in heparinized blood samples from patients with non-Hodgkin's lymphoma or acute lymphoblastic leukemia and in sera of patients with hereditary stomatocytosis [48]. Reporting [Na]_SW_, not [Na]_S_, would significantly clarify this topic [6,46,48]. This will require changes in the algorithms of the autoanalyzers, which, as noted earlier, estimate [Na]_S_ currently, plus large studies to determine the normal range of [Na]_SW_ [48].

Method of classifying hyponatremias

The classification of a case of hyponatremia in the proper tonicity category includes the following steps: (1) Measurement of the serum osmolality, which expresses total solute concentration in serum water. Osmolality is measured by a colligative property, e.g., depression of the freezing point of the sample measured, and is reported in mOsm/kg H_2_O. (2) Calculation of the serum osmolarity, which represents an estimate of the total solute concentration in serum, not in serum water. Serum osmolarity is computed, not measured directly, and is expressed in mOsm/L or mmol/L. Many formulas have been proposed for expressing serum osmolarity [51]. The simplest formula, $Osmolarity = 2x[Na]S + ([Glu]S) + serum urea$, with all concentrations expressed in mmol/L [52], is considered the preferred estimate of the serum osmolarity [53]. (3) Calculation of the osmol gap. Serum osmolarity, expressed as $(serum osmolality) x SWC$, where SWC has a value lower than one, is always less than serum osmolality. The osmol gap is the difference between measured serum osmolality and calculated serum osmolarity [6,54]. Normal values of the osmol gap using the Smithline and Gardner formula for osmolarity [52] range up to 12 mmol/L. Osmol gaps above this value merit investigation for pseudohyponatremia, the presence of exogenous solutes distributed in body water, e.g., ethanol, or in the extracellular compartment, e.g., mannitol, or the presence in the serum of excessive amounts of endogenous solutes other than sodium salts, glucose, and urea, e.g., in the sick cell syndrome [55]. (4) Calculation of the serum tonicity by the formula $2x[Na]S + [Glu]S$ expressed in mmol/L [2].

**Figure 1:**
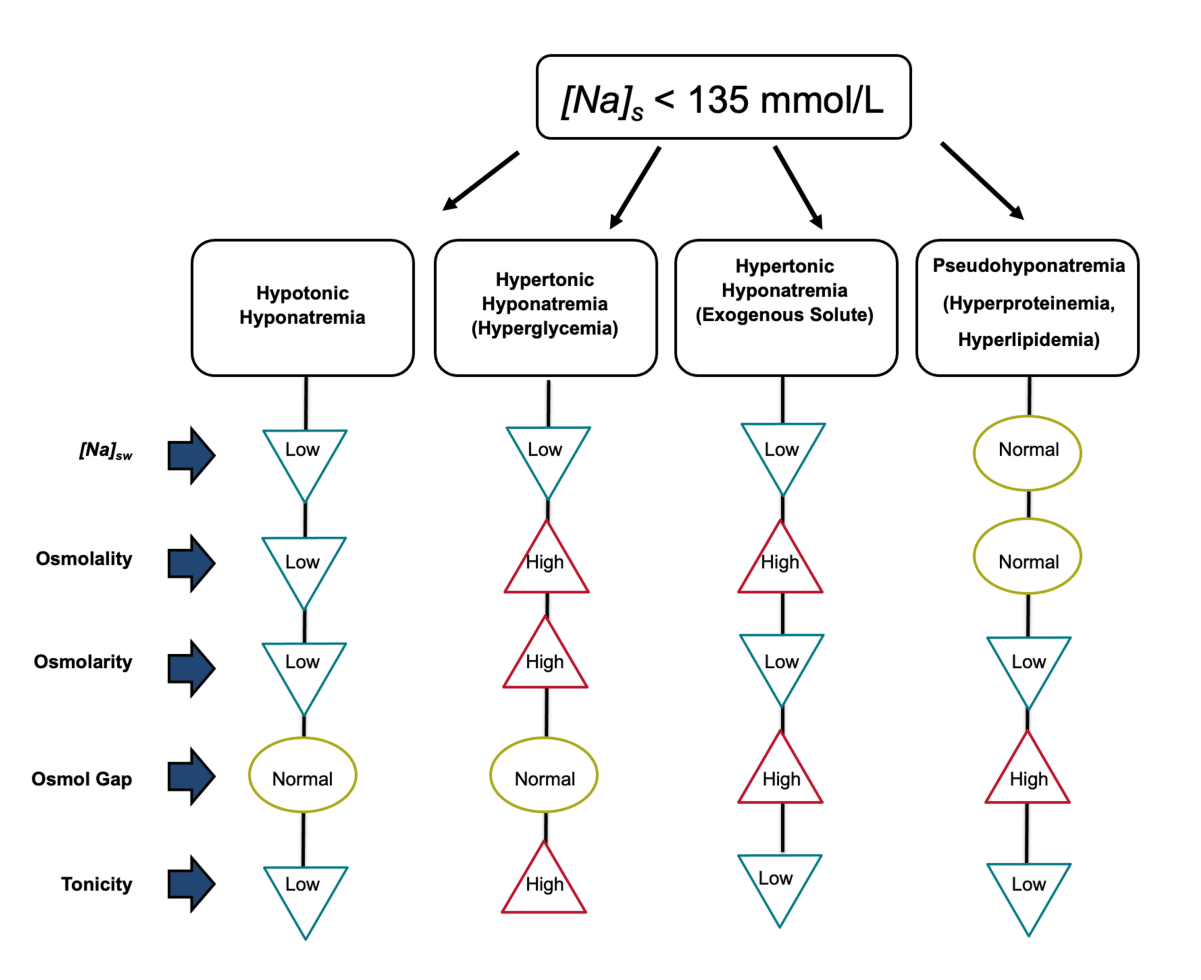
Characteristic features of the pure forms of the categories of hyponatremia. The features of this figure address measurement of [Na]_S_ by a method requiring pre-measurement dilution of the serum sample and computation of serum osmolarity and tonicity by the formulas shown in the text. The low [Na]_S_ values result in low osmolarity and tonicity values in hypertonic hyponatremia from excess exogenous solutes and in pseudohyponatremia and may cause misclassification of these categories as hypotonic hyponatremias. The features of this figure were conceived by Antonios Tzamaloukas and created by Dr. Brent Wagner.

The primary use of tonicity is for estimating cell volume in cases of hyperglycemia. At presentation with hyperglycemia, cell volume is reduced when the value of tonicity calculated by this formula is above the normal range of 275-295 mmol/L [2]. When the tonicity value is within the normal range or, in rare cases, below normal, cell volume is respectively normal or enlarged. To prevent neurological manifestations from rapid changes in brain cell volume during correction of hyperglycemia, serum tonicity, not serum osmolality or osmolarity, should be monitored [38]. Solutes distributed in body water contribute to osmolality and osmolarity, but not to tonicity. For example, urea, which is included in the formula for serum osmolarity, is excluded from the calculation of tonicity because it is distributed in body water. When its extracellular concentration changes slowly, it tends to equilibrate across cell membranes without causing a change in cell volume. Figure 1 presents the characteristic features of pure forms of the categories of hyponatremia.

Pitfalls from misclassification of hyponatremias

The processes of diagnosis and management of hyponatremias have significant pitfalls [56]. Severe errors may result from misclassification of hyponatremia. Misclassification in the two categories of hyponatremia with a high osmol gap, depicted in Figure 1, has resulted in serious complications. Misclassification and treatment as hypotonic hyponatremia of hypertonic hyponatremia secondary to excess of mannitol resulted in severe neurological manifestations [31], and of pseudohyponatremia as hypotonic hyponatremia resulted in death in one reported case [57].

Several publications have used incorrect terms for expressing total solute concentration in serum, e.g., osmolality for the sum of [Na]_S_ plus [Glu]_S_ plus serum urea. More importantly, wrong labeling of a category of hyponatremia may produce serious treatment errors. Hypertonic hyponatremia secondary to hyperglycemia has been falsely labeled as pseudohyponatremia in several published reports. Risk of inappropriate treatment occurs in this misclassification, especially in cases of combined hypertonic hyponatremia from extracellular solute (glucose) gain and pseudohyponatremia [48]. Placing this combination of hyponatremias under one category fails to appreciate that they are two distinct conditions requiring different types of treatment.

Simultaneous presence of dysnatremias secondary to different pathophysiological processes or dysnatremias and other solute abnormalities is another category of potential errors. The features of the pure forms of hyponatremia depicted in Figure 1 are masked by the presence of other conditions affecting the relation between body solutes and body water. It is important to determine whether one is dealing with a pure case of hyponatremia or a combined case before starting treatment. A few examples are shown below.

In hypotonic hyponatremia developing in azotemic patients, serum osmolality and osmolarity may be low, normal, or high, depending on the serum urea concentration, while serum tonicity is low and the osmol gap is either normal or slightly enlarged [58]. In hypotonic hyponatremia of individuals with high ethanol blood levels, serum osmolality may be low, normal, or high, depending on the serum level of ethanol, while serum osmolarity and tonicity computed by the formulas shown previously are low, reflecting the low [Na]_S_, and serum osmol gap is invariably high, reflecting the serum ethanol level [59].

As noted earlier, infusion of immunoglobulin preparations may result in the combination of hypertonic hyponatremia and pseudohyponatremia, and irrigation of operative fields with glycine or sorbitol solutions may result in the combination of hypotonic and hypertonic hyponatremias. Severe hyperglycemic syndromes are often present with combinations of several dysnatremias. Hypertonic hyponatremia is present in all hyperglycemic episodes, regardless of the presenting [Na]_S_. In hyperglycemic episodes with osmotic diuresis, water loss in excess of sodium plus potassium increases the fraction $(ETBNa + ETBK)/TBW$, [Na]_S_, and [Na]_Cor_ above the value predicted by glucose gain [40], while thirst and fluid intake result in hypotonic hyponatremia, indicated by [Na]_Cor_ below the normal range of [Na]_S_, in a small number of these patients [41]. Patients with hyperglycemic syndromes may also have pseudohyponatremia from hyperlipidemia [60]. Their treatment should address pseudohyponatremia, hypertonic hyponatremia, and the effect of osmotic diuresis or thirst. Serum osmolality should be measured, and osmolarity should be calculated for all hyperglycemic episodes. When [Na]_S_ is measured by indirect potentiometry and the calculated osmol gap is high, a higher [Na]_S_ value obtained by direct potentiometry measurement, bringing the osmol gap within its normal range, confirms the presence of pseudohyponatremia. The [Na]_Cor_ should be computed using the [Na]_S_ value from the direct potentiometry measurement. To obtain a normal [Na]_S_ at euglycemia, the composition of the replacement solution should be determined using this [Na]_Cor_ value, not the value of tonicity at hyperglycemia [41].

The potential association of hypertonic hyponatremia and pseudohyponatremia, resulting from hypertriglyceridemia, should be studied in every case of hyperglycemia. As indicated in the previous session, a high osmol gap would suggest pseudohyponatremia, and as indicated in the session on pseudohyponatremia, measuring [Na]_S_ by direct potentiometry would provide an appropriate correction. Another way of providing a correction for pseudohyponatremia is by estimating the SWC when the serum concentrations of proteins and lipids are known. Several formulas estimating the SSC and the SWC using serum proteins and lipids have been reported [48]. For example, Waugh reported the following formula for estimating SWC: $100xSWC = 99.1 - 0.73x[SP] - 1.03x[SL]$, where [SP] is serum proteins in g/dL of serum and [SL] is serum lipids in g/dL of serum [46]. A hypothetical example using the Waugh formula is provided below. The formulas for calculating serum osmolarity, tonicity, and [Na]_Cor_ are shown in the sections on hypertonic hyponatremia and methods of classifying hyponatremias. Note that the value of [Na]_S_ if SWC were 0.93 is required for the proper computation of the other tonicity parameters because the algorithms of the autoanalyzers of clinical laboratories compute [Na]_S_ using an SWC of 0.93 and the normal ranges of serum osmolarity, osmol gap, and tonicity were determined assuming a [Na]_S_ at an SWC of 0.93.

A patient presents with a history of polyuria, thirst and fluid consumption, and weight loss for 10 days; recent mental changes; profound hypotension; tachycardia; [Glu]s = 111.2 mmol/L (2002 mg/dL); measured by indirect potentiometry. [Na]s = 114 mmol/L; serum urea = 30 mmol/L (serum urea nitrogen 84 mg/dL); and serum osmolality = 402 mOsm/kg H_2_O. Calculated serum values are as follows: Osmolarity = 2x114 + 111.2 + 30 = 369.2 mOsm/L; osmol gap = 402 - 369.2 = 32.8 mmol/L; tonicity = 2x114 + 111.2 = 339.2 mOsm/L; [Na]_Cor_ = 114 + 1.6x(111.2 - 5.6)/5.6 = 144.2 mmol/L. This computed [Na]_Cor_ suggests that the losses of water and solutes determining the tonicity were equal, and the replacement solutions retained in the body should be isotonic. Measurements in serum produced very high concentrations of both lipids, consistent with the hyperglycemic syndrome, and proteins, consistent with hemoconcentration from osmotic diuresis. Based on the serum protein and lipid concentrations, an SWC of 85% (0.85) is computed by the Waugh formula, indicating a [Na]_SW_ of 114/0.85 = 134.1 mmol/L, which at an SWC of 0.93 would produce a [Na]_S_ of 134.1x0.93 = 124.7 mmol/L. The following calculations are performed in serum values: Osmolarity = 2x124.7 +111.2 + 30 = 390.6 mOsm/L; osmol gap = 402 - 390.6 = 11.4 mOsm/L; tonicity = 2x124.7 + 111.2 = 360.6 mOsm/L; and [Na]_Cor_ = 124.7 + 1.6x(111.2 - 5.6)/5.6 = 154.9 mmol/L, suggesting that loss of water was proportionally greater than the losses of solutes, creating the need for hypotonic replacement solutions.

## Conclusions

Hyponatremias should be classified as hypotonic, hypertonic, or pseudohyponatremias. Identification of the category or combination of categories of dysnatremias should be completed prior to starting treatment. It is critical for clinicians managing patients with a reported low [Na]_S_ to know how to check for the presence of any of the three categories of hyponatremia or their combinations, to understand the effects of these categories on intracellular volume, and to diagnose and manage combinations of dysnatremias.
